# Co-occurring hydrocephalus in autism spectrum disorder: a Danish population-based cohort study

**DOI:** 10.1186/s11689-021-09367-0

**Published:** 2021-04-28

**Authors:** Tina Nørgaard Munch, Paula Louise Hedley, Christian Munch Hagen, Marie Bækvad-Hansen, Jonas Bybjerg-Grauholm, Jakob Grove, Merete Nordentoft, Anders Dupont Børglum, Preben Bo Mortensen, Thomas Mears Werge, Mads Melbye, David Michael Hougaard, Michael Christiansen

**Affiliations:** 1 Department of Epidemiology Research, Statens Serum Institut, Copenhagen, Denmark; 2 Department of Neurosurgery, Copenhagen University Hospital, Blegdamsvej 9, DK-2100 Copenhagen, Denmark; 3 Department of Clinical Medicine, University of Copenhagen, Copenhagen, Denmark; 4 Department for Congenital Disorders, Statens Serum Institut, Copenhagen, Denmark; 5 The Lundbeck Foundation Initiative for Integrative Psychiatric Research, iPSYCH, Aarhus, Denmark; 6 Department of Biomedicine – Human Genetics, Aarhus University, Aarhus, Denmark; 7 Bioinformatics Research Centre, Aarhus University, Aarhus, Denmark; 8 Center for Genome Analysis and Personalized Medicine, Aarhus, Denmark; 9 Mental Health Centre, Capital Region of Denmark, Copenhagen, Denmark; 10 Department of Biomedicine and the iSEQ Centre, Aarhus University, Aarhus, Denmark; 11 Centre for Register Research, Institute of Economics, Aarhus University, Aarhus, Denmark; 12 Department of Medicine, Stanford University School of Medicine, Stanford, CA USA; 13 Department of Biomedical Science, University of Copenhagen, Copenhagen, Denmark

**Keywords:** Autism spectrum disorder, Hydrocephalus, Epidemiology, Cohort, Congenital

## Abstract

**Background:**

The association between autism spectrum disorder and hydrocephalus is not well understood, despite demonstrated links between autism spectrum disorder and cerebrospinal fluid abnormalities. Based on the hypothesis that autism spectrum disorder and hydrocephalus may, at least in some cases, be two manifestations of a shared congenital brain pathology, we investigated the potential association between autism spectrum disorder and hydrocephalus in a large Danish population-based cohort.

**Methods:**

Patients and controls were obtained from the Lundbeck Foundation Initiative for Integrative Psychiatric Research iPSYCH2012 case-cohort, which includes all patients with selected psychiatric disorders born in Denmark 1981–2005 along with randomly selected population controls (end of follow-up, December 31, 2016). The associations between individual psychiatric disorders and hydrocephalus were estimated using binary logistic regression with adjustment for age and sex.

**Results:**

The cohort consisted of 86,571 individuals, of which 14,654 were diagnosed with autism spectrum disorder, 28,606 were population controls, and the remaining were diagnosed with other psychiatric disorders. We identified 201 hydrocephalus cases; 68 among autism spectrum disorder patients and 40 among controls (OR 3.77, 95% CI 2.48–5.78), which corresponds to an absolute risk of 0.46 % (i.e. approximately one in 217 children with autism spectrum disorder had co-occurring hydrocephalus). The autism spectrum disorder-hydrocephalus association was significant over the entire subgroup spectrum of autism spectrum disorder.

**Conclusions:**

Given the considerable risk of hydrocephalus among patients with autism spectrum disorder, we suggest that patients with autism spectrum disorder should be evaluated for co-occurring hydrocephalus on a routine basis as timely neurosurgical intervention is important. Likewise, attention must be paid to traits of autism spectrum disorder in children with hydrocephalus. The results of this study call for future investigations on a potential shared aetiology between hydrocephalus and autism spectrum disorder, including the role abnormal CSF dynamics in the pathogenesis of autism spectrum disorder.

**Supplementary Information:**

The online version contains supplementary material available at 10.1186/s11689-021-09367-0.

## Background

In Denmark, the setting for this study, the prevalence of autism spectrum disorder in children is reported to be 68.5/10,000 [1] with a male to female ratio of 4:1 [2]. Characterized by impairments in social interaction, communication, and repetitive or restrictive behaviors [3], autism spectrum disorder is associated with a plethora of comorbidities, often neurodevelopmental in nature [4, 5]. Shen et al. showed in different MRI studies that children with autism spectrum disorder, up to preschool age, consistently exhibit increased volumes of CSF in the subarachnoid space, as compared to other children, a condition also known as external hydrocephalus [6–9].

Hydrocephalus is an aetiologically heterogeneous neurological condition with the broad definition excessive amount of intracranial CSF relative to the brain volume, which occurs in 1.08/1,000 children up to 2 years of age in Denmark [10]. A relatively common presentation is the Benign Enlargement of the Subarachnoid Space (BESS) or benign external hydrocephalus, that is asymptomatic and usually resolves spontaneously before the child reaches the age of 2 years [11, 12]. The signs and symptoms which most hydrocephalus patients present with do not necessarily include the well-known acute symptoms of increased intracranial pressure or obvious increased head circumference. They may present with normal or only slightly increased head circumference, and variable degrees of psycho-motoric delay or failure to thrive during infancy. When neurosurgical treatment is required, they undergo endoscopic fenestration of the third ventricle or insertion of a ventriculoperitoneal shunt.

Although children with hydrocephalus needing surgical treatment remarkedly often display traits compatible with autism spectrum disorder, only one population-based study has investigated this potential association. Thus, Lindquist et al. reported that autism was present in nine out of the 107 children with hydrocephalus (13%) born in western Sweden during the period 1989–1993 [13]. However, this potential association has not been investigated in larger cohorts before. Most likely, this is due to the relatively low prevalence of both diseases, which would require very large study populations. The access to the unique iPSYCH cohort data enabled us to investigate this question in a large population-based cohort including 14,000+ individuals with autism spectrum disorder.

Based on the hypothesis that autism spectrum disorder and hydrocephalus may, at least in some cases, be two manifestations of a shared congenital brain pathology, we investigate (1) the association between autism spectrum disorder and hydrocephalus in a large Danish population-based cohort and (2) the associations between hydrocephalus and the psychiatric disorders: schizophrenia, bipolar disorder, major depressive disorder, autism spectrum disorder, and attention-deficit hyperactivity disorder (ADHD).

## Methods

### Data sources

This register-based case-cohort study is a sub-study of the Lundbeck Foundation Initiative For Integrative Psychiatric Research (iPSYCH, http:www.iPSYCH.au.dk) and combines data from The National Patient Register [14], The Danish Neonatal Screening Biobank [15], and the Danish Psychiatric Central Research Register [16]. The registers contains information about all in-patient admissions since 1969 and out-patient visits from 1995 onwards. The data were linked on an individual level using the unique personal identification number from the Danish Civil Registration System [17].

### Cohort

Patients and controls were obtained from the Lundbeck Foundation Initiative for Integrative Psychiatric Research iPSYCH2012 case-cohort [18], which comprises information on individuals with psychiatric disorders (*n* = 57,875) as well as a randomly sampled control cohort from the same source population as the psychiatric cases (*n* = 28,606). Thus, the controls represent the background population. Cases and the population cohort were selected from a study base of 1,472,762 singleton births between May 1, 1981, and December 31, 2005, who were alive and resided in Denmark at their first birthday. End of follow-up was December 31, 2016. Hence, the cohort members were followed for at least 11 years and up to 34 years and 8 months. Cases were defined as all individuals registered with a diagnosis of five selected psychiatric disorders: schizophrenia, bipolar disorder, major depressive disorder, autism spectrum disorder, and attention-deficit and hyperactivity disorder.

### Variables

The outcome variables were a registered diagnosis of at least one of the psychiatric disorders and/or a registered diagnosis of hydrocephalus. The ICD-8 system was used from 1981 to 1994, but it was replaced by the ICD-10 system from 1995 and onwards.

### Ascertainment of psychiatric disease

The study base was linked, through the personal identification number, to the Danish Psychiatric Central Research Register to obtain information about psychiatric disorders, defined by the ICD-10 codes: F20 schizophrenia, F30–F31 bipolar disorder, F32–F39 major depressive disorder, F84 autism spectrum disorder, or F90 attention-deficit hyperactivity disorder (ADHD). ASD was furthermore divided into the subgroups defined in the ICD-10 system: F84.0 childhood autism, F84.1 atypical autism, F84.5 Asperger’s syndrome, F84.8 other pervasive developmental disorder, and F84.9 pervasive developmental disorder, unspecified. The ASD diagnosis in the Danish Psychiatric Central Register has been validated [19].

In cases where more than one condition was diagnosed, the diagnoses were prioritized with respect to their rank in the ICD-10 code system. Thus, for this study, each individual was only captured within one diagnostic category, according to the following order: schizophrenia > bipolar disorder > major depressive disorder > autism spectrum disorder > attention-deficit and hyperactivity disorder. Therefore, the case numbers of the psychiatric disorders are somewhat different from the case numbers of the iPSYCH cohort described by Pedersen et al., in which the diagnoses were not ranked, and the cohort members might have more than one diagnosis [18].

### Ascertainment of hydrocephalus

The study base was linked, through the personal identification number, to The National Patient Register. Hydrocephalus cases were identified using the wide range of existing hydrocephalus diagnoses. In case of more than one diagnosis, the following ranking order was used, according to specificity: G91.1 obstructive hydrocephalus; 74200, 74201, 74208, 74209, Q038x, and Q039 congenital hydrocephalus; G91.0 communicating hydrocephalus; G91.3, 34794 post traumatic and acquired hydrocephalus; and G91.8, G91.9 hydrocephalus, not specified. As the ICD-10 system is the most recent and most specific classification used during the study period, the ICD-10 diagnoses were ranked higher than the ICD-8 diagnoses.

We deliberately did not include hydrocephalus related to birth trauma (P10x, P91.7) and spontaneous perinatal intracranial haemorrhage (P52), because they have such obvious acquired aetiologies. We chose to include post traumatic hydrocephalus because hydrocephalus/ventriculomegaly is a relatively common incidental finding on brain CT scans performed at emergency units in patients who suffered mild to moderate head trauma. Thus, incidental findings of subclinical congenital hydrocephalus may be interpreted as post traumatic hydrocephalus. Only few patients actually develop symptomatic hydrocephalus after severe head trauma (3.56%) [20], and those who do may hold genetic predisposing factors.

### Statistical analysis

The risk of hydrocephalus among patients with psychiatric disorders compared to background population controls were estimated as odds ratios (ORs) using logistic regression analysis including age and sex as covariates, both in the primary analyses and sub-analyses. A proportion test was performed to test for differences between groups with the autism spectrum disorder-hydrocephalus association as reference. A *p*-value of 0.05 was considered significant and Bonferroni correction for multiple testing was performed where appropriate. For groupings with 30+ hydrocephalus-positive cases, a sampling distribution of the test statistic of the logit model of the hydrocephalus status was resampled 25K times and the coefficients for hydrocephalus extracted from the logit model and compared to the observed. The *p*-values were computed as two-sided. As the number of hydrocephalus-positive observations within the groupings was relatively low, the statistical tests were confirmed by a Fisher’s exact test (data not shown). Wilcoxon rank sum test was used to compare median age at diagnosis of hydrocephalus among controls and each of the psychiatric disorders.

## Results

Basic characteristics of the cohort are presented in Table 1, including the numbers of patients with each of the five psychiatric disorders, median age at diagnosis, and by the end of follow-up. Among the 86,481 cohort members, we identified 201 hydrocephalus cases, distributed as follows: 40 among the 28,606 population controls, 68 among the 14,654 patients with autism spectrum disorder (OR 3.77, 95% CI 2.48–5.78, *p*≤4 × 10^−5^, and 37 among the 13,901 patients with ADHD (OR 1.91, 95% CI 1.20–3.04, *p*=0.07), as presented in Table 2. The remaining 56 cohort members with hydrocephalus were diagnosed with either major depressive disorder, bipolar disorder, or schizophrenia, but no significant associations were found between hydrocephalus and these disorders. Nevertheless, the OR for hydrocephalus among the 5229 cohort members with schizophrenia was as high as 2.01, 95% CI 1.00–3.83, *p*=0.51 so this association may have reached statistical significance with higher numbers. The strong association between hydrocephalus and autism spectrum disorder was supported by permutation testing (Supplementary figure 1). Additionally, we performed a proportion test, as presented in Table 2. The results supported that the autism spectrum disorder-hydrocephalus association was significantly different from the associations between the other psychiatric disorders and hydrocephalus, although the schizophrenia-hydrocephalus association was close to not being significantly different (*p*=0.047).

**Table 1:** Basic characteristics of the study population

	*N*	Median age in years, Dec 2016^b^ (interquartile range)	Male (%)	Median age at diagnosis in years (interquartile range)
No psychiatric disease (controls)	28,606	22.6 (16.9–28.6)	50.4	Not relevant
Schizophrenia	5242	28.6 (25.3–32.2)	53.3	21.2 (18.9–24.0)
Major depressive disorder	22,868	28.8 (25.0–32.2)	31.9	20.0 (17.0–23.3)
Bipolar disorder	2300	29.7 (26.5–32.8)	35.6	22.9 (20.0–26.5)
Autism spectrum disorder	14,564	19.8 (16.2–23.9)	80.9	9.6 (6.4–13.3)
Childhood autism	4447	17.9 (14.6–21.6)	81.4	7.3 (4.8–12)
Atypical autism	1803	19.7 (16.2–23.6)	75.8	10.8 (7.1–14.4)
Asperger’s syndrome	4405	21.7 (17.7–25.9)	83.6	11.5 (8.6–14.9)
Other PDD^a^	1868	20.7 (16.9–24.5)	80.9	10.5 (7.6–13.5)
Unspecified PDD	2131	19.7 (15.9–24.5)	78.5	9.5 (6.6–13.2)
Attention-deficit hyperactivity disorder	12,901	21.2 (16.8–25.7)	75.2	11.4 (8.4–17.0)

^a^Pervasive developmental disorder.

^b^End of follow-up

**Table 2:** Associations between hydrocephalus and five psychiatric disorders

Condition	Hydrocephalus (*N*=201)		Logistic regression adjusted for age and sex			Proportion test between groups
	Present ***N***	Absent ***N***	OR	95% CI	***p***-value^**a**^	***p***-value
No psychiatric disease (controls)	40	28,566	1	Reference	Reference	-
Schizophrenia	13	5229	2.01	(1.00–3.83)	0.5	0.047
Major depressive disorder	40	22,828	1.22	(0.75–1.99)	1.0	<5 ×10^−7^
Bipolar disorder	3	2297	1.06	(0.25–3.08)	1.0	0.03
Autism spectrum disorder	68	14,586	3.77	(2.48–5.78)	<4 × 10^−5^	Reference
Attention-deficit and hyperactivity disorder	37	12,864	1.91	(1.20–3.04)	0.07	0.02

^a^Bonferroni corrected for multiple tests (here five)

Furthermore, we investigated the associations between each of the clinical subgroups of autism spectrum disorder and hydrocephalus, as presented in Table 3. In all five subgroups, we found a significant association with hydrocephalus: childhood autism (OR 3.71, 95% CI 2.08–6.51), atypical autism (OR 5.58, 95% CI 2.81–10.4), Asperger’s syndrome (OR 2.88, 95% CI 1.49–5.31), other pervasive developmental disorders (OR 3.35, 95% CI 1.41–7.02), and pervasive developmental disorders, unspecified (OR 3.46, 95% CI 1.53–7.01).

**Table 3:** Associations between hydrocephalus and clinical subgroups of autism spectrum disorders

Condition	Hydrocephalus		Logistic regression adjusted for age and sex		
	Present ***N***	Absent ***N***	OR	(95% CI)	***p***-value^**a**^
No psychiatric disease (controls)	40	28,566	1	Reference	Reference
Childhood autism	23	4424	3.71	(2.08–6.51)	3×10^−5^
Atypical autism	13	1790	5.58	(2.81–0.40)	1.0×10^−6^
Asperger’s syndrome	15	4390	2.88	(1.49–5.31)	0.005
Other pervasive developmental disorder	8	1860	3.35	(1·41–7·02)	0.014
Pervasive developmental disorder, unspecified	9	2122	3.46	(1·53–7·01)	0.006

^a^Bonferroni corrected for multiple tests (here five)

### Sub-analyses

In a sub-analysis, we subdivided the ICD-codes for hydrocephalus into five groups to see if the association between ASD and hydrocephalus was driven by a particular subgroup of hydrocephalus. The results are presented in Table 4. Only the subgroups obstructive hydrocephalus (OH) and congenital hydrocephalus (CH) reached sufficient numbers for estimating odd’s ratios. The results showed that the associations between autism spectrum disorder and both of the groups were significant; OR ASD-OH 5.89, 95% CI 3.02–12.10 (*p*<4.0E−5), and OR ASD-CH 2.50, 95% CI 1.30–4.84 (*p*=0.0067).

**Table 4:** Median age at hydrocephalus diagnosis, and association with autism spectrum disorder, according to ICD-subtype distribution of hydrocephalus

Hydrocephalus diagnoses (ICD-8 and ICD-10 codes)^a^	*N* (201)	Median age at diagnosis	OR 95% CI	*P*-value
Obstructive hydrocephalus^b^ (G911)	67	8.3	5.89 (3.02–12.10)	<4.0E−5
Communicating hydrocephalus^b^ (G910, G912)	17	18.4	-	-
Congenital hydrocephalus (74200, 74201, 74208, 74209, Q038x, Q039)	79	0.8	2.50 (1.30–4.84)	0.0067
Post traumatic/acquired hydrocephalus (34794, G913)	7	2.3	-	-
Hydrocephalus, not specified^b^ (G918, G919)	31	2.7	-	-

The more recent and more specific ICD-10 system diagnoses was ranked than the ICD-8 diagnoses

^a^In case of more than one hydrocephalus diagnosis, the more specific diagnosis was ranking higher

^b^Congenital (Q), or likely to be congenital due to the low median age at hydrocephalus diagnosis and the young age of the study population (G)

**Fig. 1:**
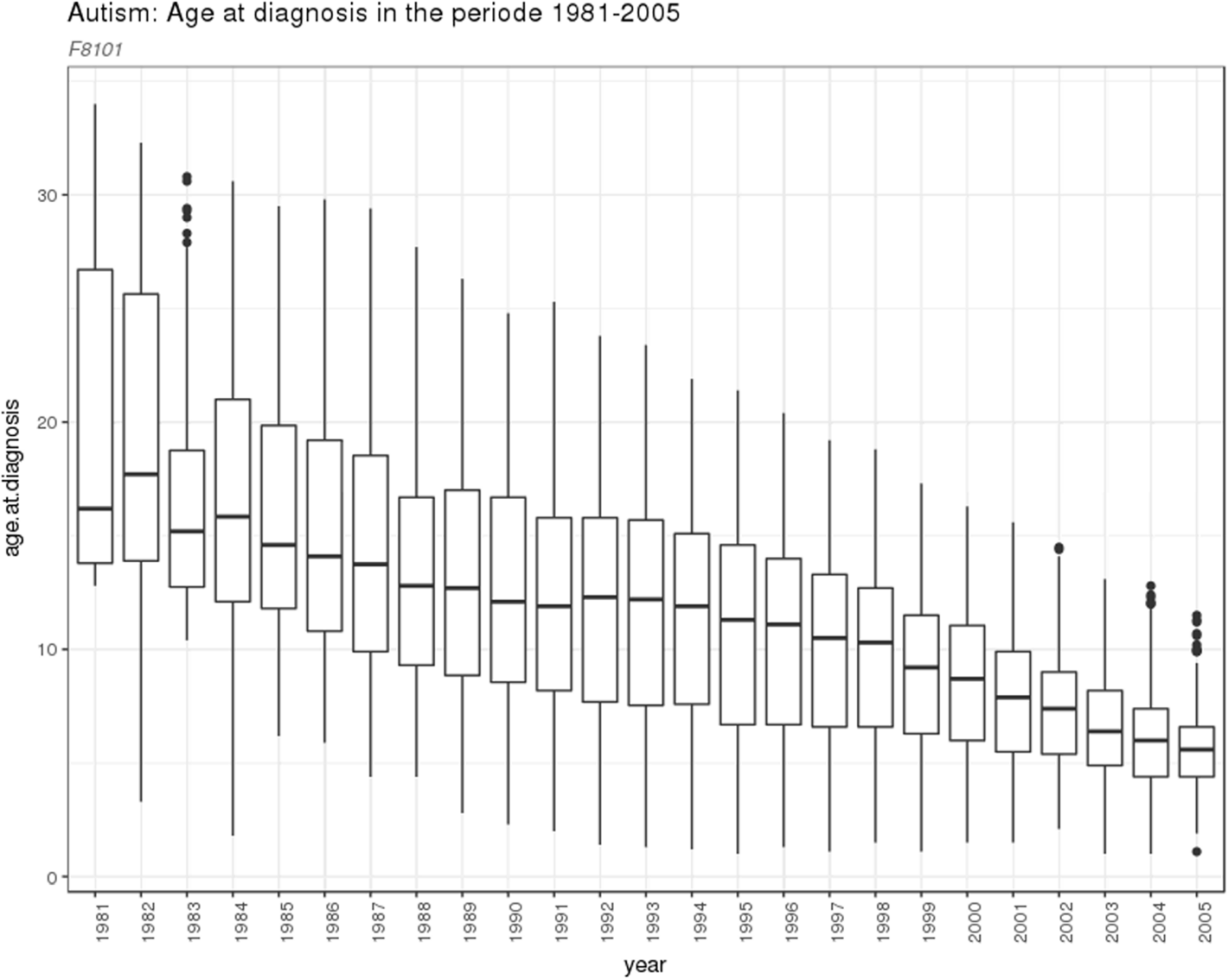
The median age at diagnosis for all cohort members with autism spectrum disorder by year of birth

The diagnostic process and the diagnostic definition of autism spectrum disorder changed significantly during the period 1981–2005, in which the cohort members were born. This meant that cohort members diagnosed with autism spectrum disorder before 1994 were not registered with an autism spectrum disorder diagnosis before the implementation of the ICD-10 system in 1995. The median age at diagnosis for all cohort members with autism spectrum disorder by year of birth are presented in Fig. 1. The decreasing age at diagnosis during the time period supports this notion.

A description of age at diagnosis and gender distribution of the hydrocephalus patients by co-occurring psychiatric disorders compared to hydrocephalus patients without psychiatric disorders are presented in Supplementary Table 1. Only patients with schizophrenia and major depressive disorder were diagnosed with hydrocephalus significantly later than the controls without co-occurring psychiatric disorders. Thus, hydrocephalus patients with autism spectrum disorder were diagnosed with hydrocephalus at a median age of 1.0 year (IQR 0.3–5.3 years), which was not significantly different from the median age of hydrocephalus diagnosis among controls without psychiatric disorders (2.3 years, IQR 0.3–12.1, *P*= 0.4).

The sequence of diagnoses in the 68 individuals with both autism spectrum disorder and hydrocephalus, as well as the subgroup of 23 individuals diagnosed with “childhood autism”, which is the autism spectrum disorder sub diagnosis presenting at the youngest age, is presented in Supplementary figure 2. The diagnosis of hydrocephalus preceded that of autism spectrum disorder in 55 cases (81%), whereas autism spectrum disorder preceded hydrocephalus in 13 cases (19%). A similar pattern was seen in the subgroup of 23 individuals with childhood autism and hydrocephalus. However, the results presented in Supplementary figure 2 must be interpreted with caution, because the sequence of diagnoses does not necessarily reflect the biological sequence of the diseases due to the delays of autism spectrum disorder diagnoses, as described above and in Fig. 1.

## Discussion

This is the first demonstration of a strong co-occurrence of autism spectrum disorder and hydrocephalus in a large population-based cohort (OR 3.77, 95% CI 2.48–5.78). This association was significant for the entire subgroup spectrum of autism spectrum disorder. The absolute risk of hydrocephalus among children with autism spectrum disorder was 0.46% (i.e., approximately one in 217 children with autism spectrum disorder had co-occurring hydrocephalus).

The observed strong association between autism spectrum disorder and hydrocephalus is in line with the findings of the, to our knowledge, only other population-based investigation of the subject matter. Thus, Lindquist et al. reported that nine out of the 107 children born with hydrocephalus in western Sweden during the period 1989–1993 were diagnosed with autism (13%) [13]. The occurrence of autism spectrum disorder among non-myelomeningocele hydrocephalus patients was as high as 20%, when actually evaluated as part of the study protocol using the Childhood Autism Rating Scale [21].

It is well known that autism spectrum disorder exhibits considerable psychiatric comorbidity, most notably with other neurodevelopmental disorders [4]. A total of 3625 of the cohort members with autism spectrum disorder in this study had co-occurring ADHD (25%), and a borderline significant association between ADHD and hydrocephalus was observed. A large-scale genetic study published in Science 2018 found evidence of a shared heritability between different psychiatric disorders, which may explain the frequently observed co-occurrence with ADHD [22].

It has previously been reported that co-occurring neurological or neurodevelopmental diagnoses are associated with later diagnosis of autism spectrum disorder [23, 24]. According to these studies, the presence of psychiatric and neurological disorders may result in masking or obscuring of core symptoms, which leads to delayed autism spectrum disorder diagnosis. Another reason can be high functioning level, as in Asperger, which may lead to later diagnosis. This may partly explain our finding of a relatively high median age for diagnosis of autism spectrum disorder diagnosis of 9.6 years. This notion is supported by the fact that the number of years to the second diagnosis was considerably longer if hydrocephalus was diagnosed first (Supplementary figure 2). Another important previously mentioned reason for the high median age is the delay of registered diagnoses of neurodevelopmental disorders for the cohort members born before the change into the ICD-10 system in 1995. This issue is addressed in the second of the sub analyses. As presented in Fig. 1, the median age at diagnosis decreased over the birth years of the cohort members from 1981 to 2015. Hence, the historical median age of diagnosis of autism spectrum disorders of 9.6 years observed in this study is not directly comparable to recent observations.

Our finding of a strong association between autism spectrum disorder and hydrocephalus may indicate either a causal association or a shared aetiology. Previous studies indicate that abnormal CSF dynamics may play a role in the development of autism spectrum disorder. Thus, Shen et al. demonstrated increased amounts of extra-axial CSF in children with autism spectrum disorder and age from 6 months to 4 years of age, regardless of familial risk [6–9]. Shen et al. concluded that “it is not clear whether increased CSF in the subarachnoid space may directly impact brain development and contribute to the pathology of psychiatric disorders such as ASD, or is an epiphenomenon that reflects another underlying aetiology” [8]. Either way, these findings, together with the strong association observed in this study, pave the way for the hypothesis that autism spectrum disorder could be associated with hydrocephalus through disturbed CSF dynamics, perhaps through shared genetic factors.

One could argue that the association observed in this study between autism spectrum disorder and hydrocephalus could also rely on a direct causal association, meaning that patients with hydrocephalus may develop autism spectrum disorder symptoms due to the brain damage caused by pressure to the periventricular structures in the brain. We cannot completely reject a causal association, but we do find it unlikely given the fact that even though the absolute risk of 0.46% is considerable from a clinical perspective, we would expect a higher proportion to be affected in case of a direct causal association.

Interestingly, we also observed a substantial number of hydrocephalus cases among patients with schizophrenia (0.25%). In fact, mild ventriculomegaly is considered a hallmark feature in particularly early onset schizophrenia together with other structural brain abnormalities indicating a neurodevelopmental aetiology [25–27]. In the elderly, schizophrenia may be associated with normal pressure hydrocephalus [28, 29].

A potential limitation of this study is the potential of surveillance bias, e.g. patients with severe psychiatric disorders may have a higher probability of undergoing brain imaging studies than background population controls and therefore incidental findings of hydrocephalus could be more likely in these patients. However, the significant differences between the association between autism spectrum disorder and hydrocephalus and the associations between hydrocephalus and the other psychiatric disorders, as presented in Table 2, argue against surveillance bias. However, one could argue that patients with autism spectrum disorder are more likely to undergo brain imaging studies than patients with the other psychiatric disorders investigated in this study. But the very strong association observed in this study (OR 3.77, 95% CI 2.48–5.78) is unlikely to be explained by incidental findings of hydrocephalus only, which generally is a rare disease seen in 1.08/1000 in children up to 2 years of age in Denmark [10]. Thus, we believe the effect of surveillance bias has limited impact on the results.

Another limitation is the potential for misclassification of diagnoses in a register-based study. However, autism spectrum disorder and hydrocephalus were the main diagnoses of interest and are two relatively distinct clinical diagnoses, so we believe the misclassification is limited. We deliberately chose to include congenital hydrocephalus and those of the acquired cases that are also considered to have a genetic predisposition, such as post traumatic or post meningitis hydrocephalus cases. We consider that environmental, causative factors, such as head trauma or meningitis, may require the interaction of a genetic factor, in order to manifest acquired hydrocephalus. This would explain why hydrocephalus after head trauma and meningitis is rare, also in our cohort (Table 4). Other common causes for secondary hydrocephalus are subarachnoid haemorrhage from intracranial aneurysms and intracerebral haematoma (stroke), but our cohort members have not yet reached the typical age for stroke as they are only up to 35 years old.

This study has several strengths, the most important is the case-cohort design, which is less prone to the biases of concern in conventional case-control studies because the cohort is randomly selected from the entire population-based birth cohort and therefore representative for the entire Danish background population. The design also minimized loss to follow-up and missing data as reporting of diagnoses for each hospital contact to The National Patient Register is mandatory. The fact that the study is based on nationwide data, not data for a specific region or hospital only, supports the external validity of the results.

## Conclusions

Irrespective of the cause of the association, our findings have clinical consequences as our data suggest that in patients with either disease, the significant risk of the presence of the other should be considered. Thus, patients diagnosed with autism spectrum disorder should be evaluated with imaging studies of the brain on a routine basis and attention must be paid for traits of autism spectrum disorder in children with hydrocephalus. Timely neurosurgical treatment of hydrocephalus when both conditions are present will improve the environment for the per se challenged brain development. The results of this study call for future investigations on a potential shared aetiology between hydrocephalus and autism spectrum disorder, including the role abnormal CSF dynamics in the pathogenesis of autism spectrum disorder.

## Supplementary Information

**Additional file 1: Figure S1.** Permutation tests of association between hydrocephalus and autism spectrum disorder. **Figure S2.** Interval between first-time diagnosis of ASD and first-time diagnosis of hydrocephalus (HC) in all cases (*n* = 68) of ASD and HC and in all cases of childhood autism (*n* = 23) and HC. **Table S1.** Age at diagnosis and gender distribution of the hydrocephalus patients by psychiatric co-diagnosis.

## Data Availability

The data that support the findings of this study are available from iPSYCH consortium, but restrictions apply to the availability of these data, which were used under license for the current study, and so are not publicly available. Data are however available from the authors upon reasonable request and with permission of the iPSYCH consortium.

## Abbreviations

ADHD: Attention-deficit and hyperactivity disorder
CSF: Cerebrospinal fluid
MRI: Magnetic resonance imaging
