# Supplementary material for: Co-occurring hydrocephalus in autism spectrum disorder: a Danish population-based cohort study
Source: J Neurodev Disord. 2021 Apr 28;13:19. doi: 10.1186/s11689-021-09367-0 (PMC8082886; doi:10.1186/s11689-021-09367-0)
Supplement: Supplementary file 1 — Additional file 1: Figure S1. Permutation tests of association between hydrocephalus and autism spectrum disorder. Figure S2. Interval between first-time diagnosis of ASD and first-time diagnosis of hydrocephalus (HC) in all cases (n = 68) of ASD and HC and in all cases of childhood autism (n = 23) and HC. Table S1. Age at diagnosis and gender distribution of the hydrocephalus patients by psychiatric co-diagnosis. [file 11689_2021_9367_MOESM1_ESM.docx]

**Supplementary material**

**Supplementary figure 1. Permutation tests of association between hydrocephalus and autism spectrum disorder.**

**A**


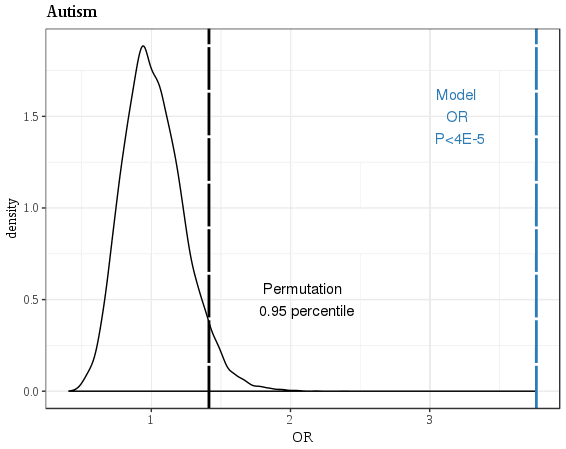


**Supplementary figure 2. Interval between first-time diagnosis of ASD and first-time diagnosis of hydrocephalus (HC) in all cases (n = 68) of ASD and HC and in all cases of childhood autism (n = 23) and HC.**

**
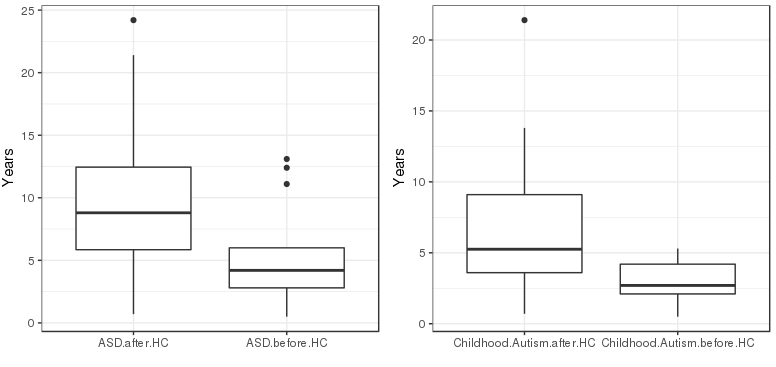
**

The diagnosis of hydrocephalus preceded that of autism spectrum disorder in 55 cases (81%), whereas autism spectrum disorder preceded hydrocephalus in 13 cases (19%). A similar pattern was seen in the subgroup of 23 individuals with childhood autism and hydrocephalus.

**Supplementary Table 1. Age at diagnosis and gender distribution of the hydrocephalus patients by psychiatric co-diagnosis.**

| Hydrocephalus (N) | Median age at diagnosis (years)  (Interquartile range) | | P ^a^ | Males (%) |
| --- | --- | --- | --- | --- |
| All cases (201) | 2.1 | (0.3 –13.7) |  | 61.0 |
| No psychiatric disease (controls) (40) | 2.3 | (0.3 –12.1) | Ref. | 42.5 |
| Schizophrenia (13) | 16.8 | (8.7 –25.6) | 0.0008 | 38.5 |
| Major depressive disorder (40) | 5.5 | (0.5 –19.2) | 0.09 | 32.5 |
| Bipolar disorder (3) | 14.2 | (7.3 –18.5) | 0.3 | 66.6 |
| Autism spectrum disorder (68) | 1.0 | (0.3 –5.3) | 0.4 | 76.5 |
| Childhood autism (23) | 1.0 | (0.4 –5.7) | 0.4 | 86.9 |
| Atypical autism (13) | 1.2 | (0.2 –3.6) | 0.5 | 84.6 |
| Asperger’s syndrome (15) | 0.7 | (0.3 –2.0) | 0.3 | 84.6 |
| Other Pervasive developmental disorder (8) | 0.9 | (0.0 – 3.4) | 0.3 | 62.5 |
| Unspecified Pervasive developmental  disorder (9) | 5.2 | (1.2 – 19.7) | 0.1 | 66.6 |
| Attention Deficit and Hyperactivity  Disorder (37) | 0.6 | (0.1 –3.4) | 0.1 | 94.5 |

^a^ Median age at diagnosis of hydrocephalus among controls were compared to each of the psychiatric disorders (Wilcoxon rank sum test).
